# 

**Published:** 1844-09

**Authors:** 


### CONTENTS.

Page

### LIBRARY.

A new Treatise on the Theory and Practice of Dental Surgery. By J. Lefoulon, Surgeon Dentist, Paris. Translated from the French, by Thomas E. Bond, Jr., M. D.... 1

### JOURNAL.

ARTICLE I.—Dissertation on the Claims of Medical Science upon the Practitioner of Dental Surgery. By Amos Westcott, M. D., D. D. S.,... 3
ARTICLE II.—Dissertation on Salivary Calculus. By William H. Dwinelle,... 32
ARTICLE III.—Observations on the Utility of Filing the Teeth. By John Harris, M. D., D. D. S., Georgetown, Ky.,... 42
ARTICLE IV.—Letter from Jesse W. Cook, M. D., D. D. S., to the Baltimore Editor,... 49
ARTICLE V.—Double Congenital Hare-lip—absence of the Superior Incisors, and their portion of Alveolar Process. By J. Marion Sims, M. D., Montgomery, Alabama,... 51
ARTICLE VI.—Baltimore College of Dental Surgery,... 57

### COLLECTANEA.

Progress of Surgery in China,... 62
Spontaneous Separation of the Anterior Portion of the Inferior Maxillary Bone—by Dr. Gambini, of Pavia,... 62
Anomalies of the Teeth,... 63
Remarkable case of Triple Dentition,... 64
On the Influence of the Epiglottis in the Formation of the Voice,... 64
Excision of the Teeth,... 65
Efforts of nature to form a Pupil at that point of the eye where it will be most useful,... 65
Constitution and By-Laws of the American Society of Dental Surgeons,... 65

### BIBLIOGRAPHICAL NOTICES.

Principles of Pathology and Practice of Medicine, by John Mackintosh, M. D., Edinburgh; with notes and additions, by Samuel George Morton, M. D.,... 71
A System of Human Anatomy, General and Special, by Erasmus Wilson, M. D., London; second American edition, by Paul Goddard, A. M., M. D.,... 71

CONTENTS.

Page

The Principles and Practice of Modern Surgery, by Robt. Druitt, Surgeon; from the third London edition, by Joshua B. Flint, M. D., M. M. S. S.,.................................... 72

### MISCELLANEOUS NOTICES.

American Society of Dental Surgeons,.............................. 73
Mississippi Valley Association of Dental Surgeons.................. 78
Singular Coincidence,.......... ................................ 80
Dr. Brown's Opening Address,....................................... 80
To Subscribers,.................................................... 80
To Correspondents,.......... ....... ............ .......... 80
University of Maryland,............................................ 80
New York University,............................................... 81
Jefferson College,............ ................ ............ 81
Gossip with our Profession,........ .......................... .. 81

